# The strength of negative plant–soil feedback increases from the intraspecific to the interspecific and the functional group level

**DOI:** 10.1002/ece3.3755

**Published:** 2018-01-26

**Authors:** Alexandra R. Bukowski, Conrad Schittko, Jana S. Petermann

**Affiliations:** ^1^ Institute of Biology Freie Universität Berlin Berlin Germany; ^2^ Institute of Biochemistry and Biology Biodiversity Research/Systematic Botany University of Potsdam Potsdam Germany; ^3^ Berlin‐Brandenburg Institute of Advanced Biodiversity Research (BBIB) Berlin Germany; ^4^ Department of Ecology and Evolution University of Salzburg Salzburg Austria

**Keywords:** *Arabidopsis thaliana* Col‐0, home‐away effect, intraspecific diversity, morphological similarities/dissimilarities of plants, plant–soil (belowground) interactions, species coexistence, taxonomic levels, trait measurements

## Abstract

One of the processes that may play a key role in plant species coexistence and ecosystem functioning is plant–soil feedback, the effect of plants on associated soil communities and the resulting feedback on plant performance. Plant–soil feedback at the interspecific level (comparing growth on own soil with growth on soil from different species) has been studied extensively, while plant–soil feedback at the intraspecific level (comparing growth on own soil with growth on soil from different accessions within a species) has only recently gained attention. Very few studies have investigated the direction and strength of feedback among different taxonomic levels, and initial results have been inconclusive, discussing phylogeny, and morphology as possible determinants. To test our hypotheses that the strength of negative feedback on plant performance increases with increasing taxonomic level and that this relationship is explained by morphological similarities, we conducted a greenhouse experiment using species assigned to three taxonomic levels (intraspecific, interspecific, and functional group level). We measured certain fitness‐related aboveground traits and used them along literature‐derived traits to determine the influence of morphological similarities on the strength and direction of the feedback. We found that the average strength of negative feedback increased from the intraspecific over the interspecific to the functional group level. However, individual accessions and species differed in the direction and strength of the feedback. None of our results could be explained by morphological dissimilarities or individual traits. *Synthesis*. Our results indicate that negative plant–soil feedback is stronger if the involved plants belong to more distantly related species. We conclude that the taxonomic level is an important factor in the maintenance of plant coexistence with plant–soil feedback as a potential stabilizing mechanism and should be addressed explicitly in coexistence research, while the traits considered here seem to play a minor role.

## 1 INTRODUCTION

The exact mechanisms maintaining species coexistence remain largely unresolved. With regard to individual plant species, not only abiotic factors (environmental conditions), but also a number of biotic factors such as intraspecific competition (Stoll & Prati, 2001), interspecific competition (Goldberg & Barton, 1992), a species’ associated soil community as well as the associated soil communities of other plant species (van de Voorde, van der Putten, & Bezemer, 2011) plays important roles for plant–plant interactions and thus for coexistence between them. Plant–soil feedback as a process potentially maintaining plant species coexistence when acting as a stabilizing mechanism (Chesson, 2000; HilleRisLambers, Adler, Harpole, Levine, & Mayfield, 2012) has received considerable attention (Bever, 2003; Bever, Platt, & Morton, 2012; Bever, Westover, & Antonovics, 1997; Ehrenfeld, Ravit, & Elgersma, 2005; Klironomos, 2002; Kulmatiski, Beard, Stevens, & Cobbold, 2008; van der Putten et al., 2013). This idea is based on the fact that a plant community influences its associated soil community, and the soil organisms have specific feedback effects on their host plants in turn (Bever et al., 1997). This soil community can contain mutualists and/or pathogens (Adewale, Aremu, & Amazue, 2012; Bever, 2003; Bever et al., 1997, 2012). In addition, abiotic mechanisms can lead to feedback effects, such as the release of allelochemical compounds by the plants or specific nutrient depletion (van der Putten et al., 2013). Negative feedback which has been reported to be more common than positive feedback, at least in experimental systems (Kulmatiski et al., 2008) may enhance species coexistence via increasing negative density dependence (i.e., as a stabilizing mechanisms sensu ChessonChesson (2000)) and thus, support the maintenance of species diversity when it is strong enough to balance out fitness differences between species (Petermann, Fergus, Turnbull, & Schmid, 2008) In contrast, positive feedback might lead to a loss of species diversity (Bever et al., 2012). The prevailing direction of plant–soil feedback may depend on a number of parameters, for example, plant functional group identity (Heinze, Bergmann, Rillig, & Joshi, 2015), plant life form (van de Voorde et al., 2011), plant abundance (Heinze et al., 2015), size‐related traits of a plant species (Heinze et al., 2015), the composition of the plant community (Kulmatiski & Kardol, 2008), and whether a plant species is native or invasive (Klironomos, 2002; Reinhart, Packer, Van der Putten, & Clay, 2003).

It has been stated that trait variation at the intraspecific as well as at the interspecific level has an influence on species coexistence (Albert, Grassein, Schurr, Vieilledent, & Violle, 2011; Bolnick et al., 2011). However, most soil feedback studies refer to the interspecific level only, comparing growth of plants on own soil with growth on soil from other species. Recently, it has been shown that plant–soil feedback may operate at the intraspecific level, that is, that there are differences in plant growth on own soil compared with growth on soil from different accessions or genotypes within the same species (Bukowski & Petermann, 2014; Liu, Etienne, Liang, Wang, & Yu, 2015). Despite indications that certain pathogens may have similar effects on closely related species (Gilbert, Briggs, & Magarey, 2015; Parker et al., 2015), it is still unclear whether there is a difference in feedback strength between the intraspecific and the interspecific levels (van der Putten et al., 2013). Indeed, there is considerable debate on whether the strength of plant–soil feedback experienced by each plant individual in a community is predictable from information on species relatedness. It has been shown that plant–soil feedback may be mediated by plant traits (Heinze et al., 2015) and that, in most cases, closely related species have similar traits (Blomberg, Garland, & Ives, 2003; Burns & Strauss, 2011; Gilbert & Webb, 2007; Webb, Gilbert, & Donoghue, 2006). Anacker, Klironomos, Maherali, Reinhart, and Strauss (2014) have related plant species relatedness to the strength of soil feedback between them, suggesting phylogeny as a major determinant of plant–soil feedback (Brandt, Seabloom, & Hosseini, 2009). On the other hand, this relationship could not be confirmed by a recent meta‐analysis (Mehrabi & Tuck, 2015).

To experimentally test whether there is indeed a relationship between the taxonomic relatedness of plants and the strength of the feedback they experience, we conducted a plant–soil feedback experiment at different taxonomic levels. The defined taxonomic levels were as follows: the intraspecific level (different accessions within a species), the interspecific level (closely related species of the same plant family), and the functional group level (species of different functional groups that are very distantly related). We measured plant–soil feedback as relative plant performance on home soil (trained by the same accession/species) *versus* away soil (trained by another accession/species), whereby positive feedback implies a better plant performance on home soil compared to away soil, and *vice versa* for negative feedback. Additionally, we compared accessions and species based on measured morphological traits in order to investigate whether morphological similarities between our accessions/species or individual traits of the accessions/species might explain the feedback effects. We hypothesized that:

1. Plant individuals experience plant–soil feedback, predominantly negative feedback, at all taxonomic levels.
2. Plant–soil feedback between accessions (i.e., at the intraspecific level) is weaker than between species (interspecific level) and functional groups (functional group level).
3. The strength of plant–soil feedback can be explained by morphological similarities between accessions/species or by individual traits.

## 2 MATERIALS AND METHODS

### 2.1 Experimental species

**Figure 1:**
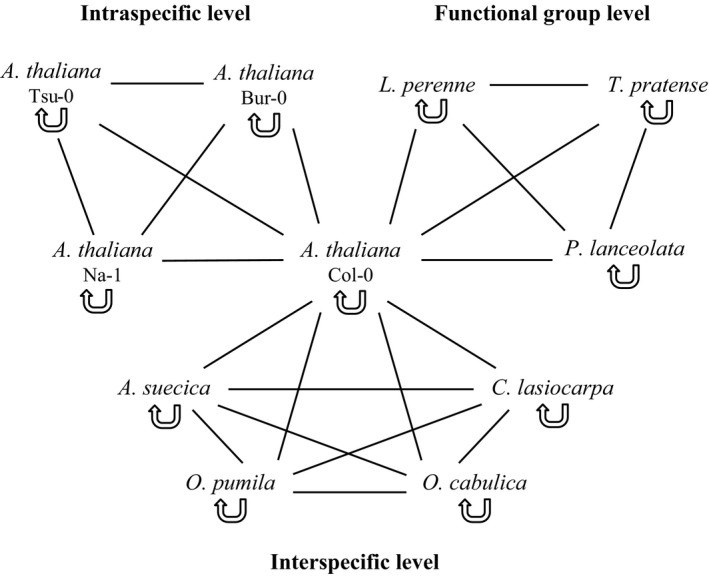
Scheme depicting the design of the experiment phase of the plant–soil feedback experiment. The experiment consisted of an intraspecific, interspecific, and functional group levels. Each accession/species was growing in home soil that had been trained by the same accession/species (indicated by the semicircular arrow) as well as in away soil that had been trained by another accession/species. Within each taxonomic level, every accession/species was growing in all possible away soil types (indicated by the connecting lines). The focal *Arabidopsis thaliana* accession Col‐0 (center) was used in all three parts of the experiment

For the plant–soil feedback experiment, a total of eleven accessions/species were used as follows: four accessions of one species for the intraspecific level, five species for the interspecific level, and four species for the functional group level (Figure 1).

*Arabidopsis thaliana* (L.) Heynh. (thale cress) was chosen as the focal plant species, therefore appearing in all three parts of our experiment (the intraspecific, the interspecific as well as the functional group level). *A. thaliana* belonging to the plant family Brassicaceae has been established as a worldwide model plant species in different fields of biology because of various advantages such as a comparatively short life cycle. To investigate plant–soil feedback at the intraspecific level, we used four natural (not genetically modified) *A. thaliana* accessions of different origins, namely Col‐0 (origin in Columbia, Missouri, USA), Tsu‐0 (Tsu, Japan), Bur‐0 (Burren, Ireland), and Na‐1 (Nantes, France). As Col‐0 is the most explored accession being used as wild type or reference accession of *A. thaliana* in most studies (Fahlgren et al., 2006; Frenkel et al., 2009; Xiao et al., 2001), we decided to use this accession in all three parts of the plant–soil feedback experiment.

To investigate plant–soil feedback at the interspecific level, we used the *A. thaliana* accession Col‐0 and four other plant species which are closely related to *A. thaliana* but do not necessarily co‐occur with *A. thaliana* or with each other. They all belong to the plant family Brassicaceae and were *Arabidopsis suecica* (Fries) Norrlin, Meddel (Swedish thale cress), *Olimarabidopsis pumila* (Stephan) Al‐Shehbaz, O'Kane & R. A. Price (dwarf rocket), *O. cabulica* (J. D. Hooker & Thomson) Al‐Shehbaz, O'Kane & R. A. Price (rock‐cress), and *Crucihimalaya lasiocarpa* (J. D. Hooker & Thomson) Al‐Shehbaz, O'Kane & R. A. Price (no English name). The taxonomy of *A. thaliana* and its close relatives has changed many times in the past (Al‐Shehbaz & O'Kane, 2002; Kiefer et al., 2014; Koch, Bishop, & Mitchell‐Olds, 1999; Price, Palmer, & Al‐Shehbaz, 1994). According to the latest classification, all species used here rate among a monophyletic group which consists of eleven tribes (Kiefer et al., 2014).

To investigate plant–soil feedback at the functional group level, we used the *A. thaliana* accession Col‐0 being a representative of the functional group of herbs not colonized with arbuscular mycorrhizal fungi. Additionally, we used three other plant species, each being a representative of a different functional group. These species are common in Central European grasslands but do not necessarily co‐occur with the other species in the experiment. These species were as follows: *Lolium perenne* L. (perennial ryegrass, plant family Poaceae) as a representative of the functional group of grasses, *Trifolium pratense* L. (red clover, plant family Fabaceae) as a representative of the functional group of legumes and *Plantago lanceolata* L. (ribwort plantain, plant family Plantaginaceae) as a representative of the functional group of herbs colonized with arbuscular mycorrhizal fungi.

Our approach of classifying species and accessions as either closely or distantly related is based on their taxonomic relatedness, disregarding phylogenetic relationships. Phylogeny may have the potential to determine species’ relatedness even more precisely; however, we were not able to construct a phylogenetic tree for our experimental accessions/species because precise phylogenetic data are not available for all *A. thaliana* accessions and its closely related species (but see Lee, Guo, Wang, Kim, and Paterson (2014) for phylogenies of some of our accessions).

### 2.2 Plant–soil feedback experiment

Following the common approach of conducting plant–soil feedback experiments (Aguilera, 2011; Hendriks et al., 2013; Kulmatiski, Beard, & Heavilin, 2012; Kulmatiski & Kardol, 2008; Petermann et al., 2008; Reinhart, 2012; van de Voorde et al., 2011), the experiment consisted of two phases. This was a training phase consisting of monocultures growing in neutral soil whereby changing its biotic and abiotic conditions in a specific way and a subsequent experiment phase using the trained soil to test its effect on a new generation of plants.

#### 2.2.1 Training phase

For the first phase, we used a substrate consisting of 70% premixed soil (standard soil and perlite, see below) and 30% inoculum which was field soil taken from an old field site near Freie Universität Berlin. Using field soil enabled us to investigate the effects of the biotic components of the soil. The field soil had been sieved (mesh size: 2 mm) in order to remove stones, roots, and other large objects. The surface‐sterilized seeds were stratified in dry condition in the refrigerator at 5°C for 4 days to ensure synchronous germination. Seeds were sown in pots (height 10 cm, diameter 11 cm) according to the following design. During the entire experiment, plants were growing in monocultures in groups of ten. For this, seeds of one accession/species were placed in a circle of ten in a pot. The monocultures of the *A. thaliana* accessions Tsu‐0, Bur‐0, and Na‐1 as well as of the species *L. perenne*,* T. pratense,* and *P. lanceolata* were replicated ten times each. For the species *A. suecica*,* O. pumila*,* O. cabulica,* and *C. lasiocarpa*, the setup was replicated twelve times each in order to have a larger amount of trained soil in the experiment phase because the interspecific level consisted of five species, whereas the intraspecific and the functional group level consisted of only four accessions/species each (Figure 1). As the focal *A. thaliana* accession Col‐0 was used in all three parts of the experiment (intraspecific, interspecific, and functional group levels), 32 replicates were necessary in order to produce enough soil for the experiment phase. In total, there were 140 pots for the training phase (6 accessions/species × 10 replicates + 4 species × 12 replicates + 1 accession × 32 replicates). Within the first days, all pots were watered from above by spraying daily. Later on, pots (on separate saucers) were watered from below three times per week only. Pots were randomized once per week. Seven weeks after sowing, all plants were harvested, and the following morphological aboveground traits were measured. For all *A. thaliana* accessions as well as for *A. suecica*,* O. pumila*,* O. cabulica,* and *C. lasiocarpa*, the rosette diameter, stem height, and number of siliques were recorded for each plant individual (stem height and number of siliques could only be measured for those plants which developed stems). We decided to incorporate these latter, reproductive traits in order to test whether unfavorable conditions due to negative plant–soil feedback effects are related to higher investment in reproduction, that is, potential dispersal. Additionally, for the *A. thaliana* accession Col‐0, five siliques per plant were collected, measured in length, and weighed so that the mean silique length as well as the total silique weight could be calculated. For the species *L. perenne*,* T. pratense,* and *P. lanceolata*, the maximum height in stretched state as well as the number of leaves was determined for each plant. Afterward, plants were dried for 5 days at 50°C to constant weight. The trained soil was homogenized by hand and stored at low temperatures until the beginning of the experiment phase.

#### 2.2.2 Experiment phase

Prior to the experiment phase, the soil was prepared as follows. As the soil trained by *L. perenne*,* T. pratense,* and *P. lanceolata* contained thick roots compared to the soil trained by the other plant species, the majority of those roots was removed by sieving (mesh size: 2 mm) to adjust the amount of roots to the soil trained by the *A. thaliana* accessions as well as by *A. suecica*,* O. pumila*,* O. cabulica,* and *C. lasiocarpa* which contained fine, thin roots only. As our hypothesis was that feedbacks are stronger between plant functional groups than at other taxonomic levels, this removal of roots from the three plant species tested at this level was assumed to only weaken this result (i.e., was a conservative decision). For the experiment phase, we used the same premixed soil as background soil as for the training phase plus an inoculum of one of the trained soil types. The premixed soil was steamed for sterilization so that possible feedback effects could be traced back to the inoculum. The proportion of inoculum was 15% by weight. The experimental design was as follows. Within each part of the experiment (intraspecific, interspecific, and functional group levels), each plant species was grown in home soil, that is, trained by the same accession/species, as well as in away soil, *that is,* trained by another accession/species. The intraspecific and the functional group levels consisted of four accessions/species, so plants of those accessions/species were growing in three different away soil types. In contrast to that, the interspecific level consisted of five species, so plants of those species were growing in four different away soil types (Figure 1). Each home treatment was replicated six times for each accession/species, whereas each away treatment was replicated three times. In total, there were 198 pots for the experiment phase (11 accessions/species × 1 home treatment × 6 replicates + 8 accessions/species × 3 away treatments × 3 replicates + 5 species × 4 away treatments × 3 replicates) with ten plants of the same accession/species per pot. This phase lasted 8 weeks. Stratification, sowing, watering, randomizing, harvesting, drying, and measuring in the experiment phase followed the same protocol as in the training phase.

### 2.3 Plant material, greenhouse conditions, and soil composition

Seeds of the *A. thaliana* accessions Col‐0, Tsu‐0, Bur‐0, and Na‐1 as well as of the species *A. suecica*,* O. pumila*,* O. cabulica,* and *C. lasiocarpa* were offsprings of adult plants that had been growing for one generation in the greenhouse after an initial order at the Nottingham Arabidopsis Stock Center (NASC). Seeds of the plant species *L. perenne*,* T. pratense,* and *P. lanceolata* were ordered at Appels Wilde Samen GmbH (Darmstadt, Germany) in September 2014. Throughout the whole experiment, plants were grown in a greenhouse at Freie Universität Berlin under long‐day conditions, *that is,* a 16‐hr light (6 a.m. to 10 p.m.), 8‐hr darkness (10 p.m. to 6 a.m.) cycle with 20°C and 18°C as day and night temperatures and a humidity of 35%. The light intensity was 120 μmol quanta m^−2^ s^−1^ powered by high‐pressure sodium lamps (Philips Powertone Son‐T Agro, 400W, 2000K; Philips GmbH Market DACH, Hamburg, Germany). For both experimental phases, we used premixed soil consisting of 84.8% standard soil (42.4% potting soil and 42.4% pricking soil of Einheitserde^®^ Classic; Einheitserde Werkverband e. V., Sinntal‐Altengronau, Germany) and 15.2% perlite (Perligran^®^ Classic; Knauf Aquapanel GmbH, Dortmund, Germany) as background soil. Properties and nutrient composition were as follows: 50% organic substances, 1.7 g/L KCl, 194.5 mg/L CaCl_2_, 189 mg/L P_2_O_5_, 267 mg/L K_2_O, pH = 5.8.

### 2.4 Statistical analyses

Data analyses were performed with the software R version 3.0.0 (R Development Core Team 2013). We calculated the strength of plant–soil feedback that each accession/species experienced as a log‐transformed ratio of aboveground biomass on home versus away soil, following the approach of Petermann et al. (2008). Using mixed‐effects models with pot number as the random effect and taxonomic level as well as accession/species identity as explanatory variables (R package nlme (Pinheiro, Bates, DebRoy, & Sarkar, 2013)), we tested whether the feedback strength changed along the taxonomic level of the feedback (tested as a continuous variable: intraspecific level = 1, interspecific level = 2, and functional group level = 3) or differed among the experimental plant accessions/species. The interaction of the main effects “accession/species identity” and “taxonomic level” could not be tested because only the *A. thaliana* accession Col‐0 was used at all three taxonomic levels.

The pairs of replicate plants on home and away soil, respectively, which were used for calculating the log‐transformed feedback ratios were selected randomly for each calculated ratio. As the numbers of home and away pots were not equal (six home pots for each accession/species, nine or twelve away pots for each accession/species), some data points of home pots were randomly used twice. To test for any effect that the original pairing might have on the results, we performed an additional bootstrap procedure for the ratio calculation by sampling with replacement for 1,000 iterations. Specifically, we calculated the log‐transformed biomass ratio for each accession/species 1,000 times and calculated 95% bootstrap confidence intervals to determine whether this ratio was significantly different from zero. We used the R package boot (Canty & Ripley, 2013; Davison & Hinkley, 1997) for the bootstrap procedure.

For determining trait similarities between accessions/species, we used the three traits “biomass,” “height,” and “fitness” measured in the training phase of our experiment as well as trait values from the literature of five categorical traits. The trait “fitness” describes the proportion of individuals per pot that produced seeds. As categorical traits from the literature we used “rosette formation,” “life form,” “association with arbuscular mycorrhizal fungi (AMF),” “nitrogen fixation (NF),” and “life span” for a further characterization of each accession/species (Table S1). With these values, we calculated dissimilarities between the accessions/species using the Gower distance (1971) as the distance measure. Gower distance has the ability to cope with mixed trait data (categorical and continuous) and missing values. To analyze the relationship between trait similarities between the accessions/species and the experienced feedback strength, we used a Mantel test with 999 permutations. This was done to account for the nonindependence in the data, caused by any given species being part of multiple pairwise species comparisons. The Mantel test was performed with the R package vegan (Oksanen et al., 2013). Further, we tested whether single traits explain variation in the feedback values by using separate linear models. Relevant R scripts are available in the Supporting information.

## 3 RESULTS

**Figure 2:**
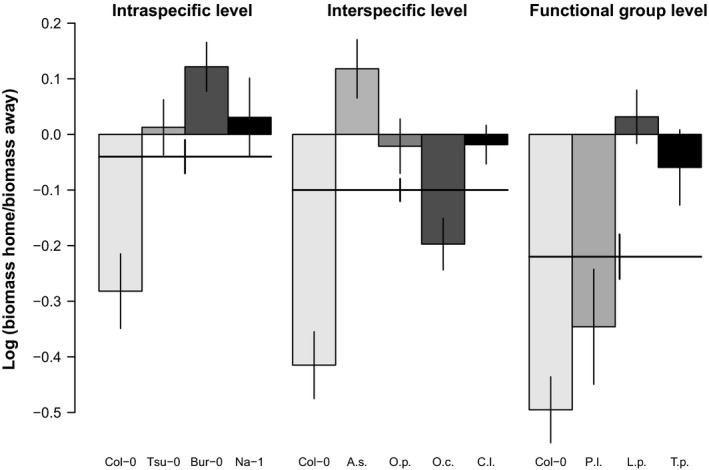
Average plant–soil feedback experienced by the *Arabidopsis thaliana* accessions Col‐0, Tsu‐0, Bur‐0, and Na‐1 (intraspecific level) as well as the species *A. suecica* (A. s.), *Olimarabidopsis pumila* (O. p.), *O. cabulica* (O. c.), *Crucihimalaya lasiocarpa* (C. l.; interspecific level), *Plantago lanceolata* (P. l.), *Lolium perenne* (L. p.), and *Trifolium pratense* (T. p.; functional group level). Feedback values are the log‐transformed ratios of the biomass of individual plants on home soils divided by the biomass of individual plants on away soils for each accession/species. Shading is used to facilitate the comparison between accessions and species, but note that the *A. thaliana* accession Col‐0 appears three times (light gray bars). Negative values indicate negative feedback, positive values indicate positive feedback. See “Statistical analyses” for detailed information regarding the calculation of feedback. Bars represent the mean ± *SE*. Bold lines show the mean ± standard error for each taxonomic level. See Table 1 for the statistical analysis

**Figure 3:**
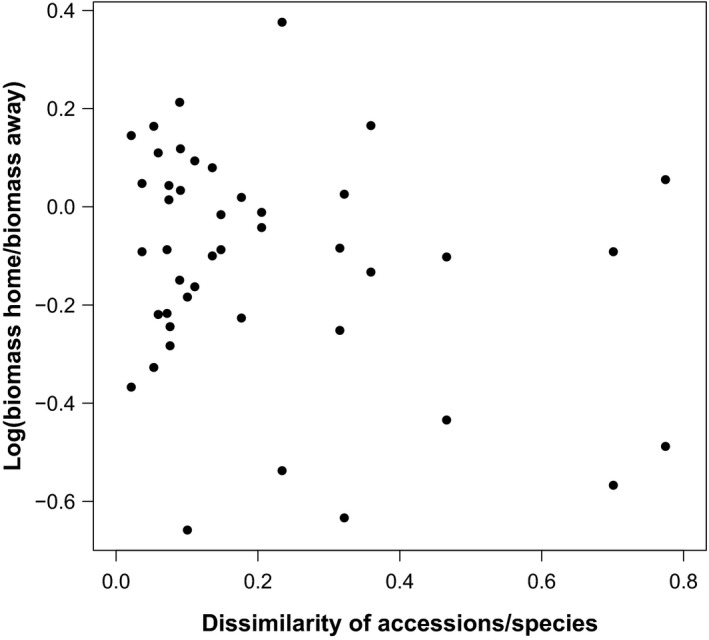
Pairwise trait dissimilarities between accessions/species show no relationship with the average plant–soil feedback of the respective accession/species pair. For determining trait dissimilarities, we used data from eight traits and calculated the Gower distance for each pair of accessions/species. The dissimilarity of accession/species pairs ranged from 0 (low dissimilarity) to 1 (high dissimilarity). To calculate the corresponding plant–soil feedback value, we used biomass data on home soils for each accession/species divided by the biomass of this accession/species on away soil that was conditioned by the corresponding accession/species from that species pair. See the Results section for the statistical analysis using a Mantel test

On average, plants at all three taxonomic levels experienced negative plant–soil feedback (Figure 2). However, the strength of negative feedback significantly increased from the intraspecific level (mean feedback for the accessions = −0.04 ± 0.03) over the interspecific level (mean feedback for the respective species = −0.10 ± 0.02) to the functional group level (mean feedback for the respective species = −0.22 ± 0.04; Figure 2, Table 1). Similarly, the focal *A. thaliana* accession Col‐0 experienced increasingly negative plant–soil feedback from the intraspecific level over the interspecific level to the functional group level (Figure 2). The strength of the feedback varied significantly between the other accessions and species (Figure 2, Table 1). The *A. thaliana* accession Bur‐0 as well as *A. suecica* showed strong positive feedback, whereas *O. cabulica* and *P. lanceolata* showed strong negative feedback and the other accessions and species experienced only weak positive or negative feedback (Figure 2). The bootstrapping showed that the pairing of the individuals on home and away soils for calculating the feedback ratios did not affect the results (Figure S1). Trait dissimilarities between accessions/species were not related to the strength of the soil feedback (Fig. 3, Mantel test with 999 permutations, Mantel *r* statistic: −.1174, *p*‐value: .68). In addition, we did not find single traits to be related to the strength of the soil feedback (Table 2).

**Table 1:** Results of the mixed‐effects model of plant–soil feedback testing the effect of taxonomic level (tested as a continuous variable: intraspecific level = 1, interspecific level = 2, and functional group level = 3) as well as accession/species identity. Pot number was used as the random effect. For details of the calculation of the feedback, see “Material and methods”

	num *df*	den *df*	*F* value	*p* Value
Intercept	1	1,017	32.17959	<.001
Taxonomic level	1	120	11.02965	.0012
Accession/species identity	10	120	7.99263	<.001

num *df*, numerator degrees of freedom; den *df*, denominator degrees of freedom.

**Table 2:** Linear model results for the effect of single traits on mean feedback values for each accession/species. “Biomass (g),” “height (cm),” and “fitness” were measured during the training phase of the experiment, whereas the categorical traits “rosette,” “life form,” “association with arbuscular mycorrhizal fungi (AMF),” “nitrogen fixation (NF),” and “life span” were extracted for each accession/species from the literature. The trait “fitness” describes the proportion of individuals per pot that produced seeds

Traits	Adjusted *R* ^2^	*F* value	*p* Value
Biomass	−.0926	0.1523	.7054
Height	−.0875	0.1957	.6687
Fitness	−.1080	0.0257	.8762
Rosette	−.0743	0.3081	.5924
Life form	−.0632	0.4054	.5402
AMF	−.0557	0.4725	.5092
NF	−.1096	0.0120	.9152
Life span	−.0557	0.4725	.5092

## 4 DISCUSSION

### 4.1 Increasing feedback strength from low to high taxonomic levels

On average, negative plant–soil feedback operated in our experiment, partly confirming our first hypothesis. The average negative feedback was stronger between functional groups (functional group level) than between species (interspecific level) and between accessions (intraspecific level), confirming our second hypothesis. In line with this, the focal *A. thaliana* accession Col‐0 experienced stronger negative feedback at the functional group level than at the interspecific level or the intraspecific level. These results indicate that the taxonomic relatedness of the involved plant species may be an important factor determining plant–soil feedback. This relationship could be driven by pathogens as agents of negative feedback which have been shown to be shared by closely related species (Gilbert et al., 2015). We also observed some (rarer) cases of positive feedback, potentially caused by potentially less specific mutualists such as mycorrhizae (Klironomos, 2002). The relationship between the relatedness of plant species and the strength of plant–soil feedback is controversially debated in the literature. A meta‐analysis which was based on results of plant–soil feedback experiments that were performed in the last 20 years could not explain the strength and direction of feedback by phylogeny (Mehrabi & Tuck, 2015). In line with this, a recent study showed that plants did not perform better when growing in soil being trained by a more distinctly related species compared to soil from a closely related species (Mehrabi, Bell, & Lewis, 2015). On the other hand, some studies have found evidence for the relationship between relatedness of plant species and strength as well as direction of plant–soil feedback (Brandt et al., 2009; Liu et al., 2012) which are confirmed by our results for the focal a *A. thaliana* accession Col‐0 as well as for the average feedback across taxonomic levels. These contrasts between studies may indicate that neither the strength nor the direction of plant–soil feedback can be explained by plant phylogeny in all cases, but that we may have to consider a number of different factors and their interplay when ascertaining the cause of observed plant–soil feedback, and relatedness between species is certainly one of them.

### 4.2 Direction and strength of plant–soil feedback vary among accessions and species

Many of the accessions and species experienced plant–soil feedback, partly confirming our first hypothesis. However, the individual accessions and species differed in the direction and strength of the feedback. With regard to intraspecific plant–soil feedback, we had formerly shown that *A. thaliana* accessions differ in the strength and direction of experienced feedback (Bukowski & Petermann, 2014). The same accessions in our experiment showed different feedback strengths and directions compared to this earlier study. However, in the earlier study, the away soil types had been trained by different accessions, likely explaining the different feedback values. Unfortunately, our data cannot be compared directly to the earlier study because of different growth conditions (mainly different substrate). Future studies on intraspecific plant–soil feedback may also incorporate different genotypes of members of different functional groups to test how prevalent these effects are.

Regarding the functional group level, our results are partly consistent with other studies which found *P. lanceolata*,* L. perenne,* and *T. pratense* to suffer from negative feedback (Bever, 2002; Harrison & Bardgett, 2010; Hendriks et al., 2015; Klironomos, 2002; Petermann et al., 2008). Furthermore, in contrast to our results, it has been shown that plants perform better in soil having been trained by species from the functional group of herbs than in soil trained by grass species due to a depletion of potassium in grass‐trained soil (Bezemer et al., 2006). However, comparing absolute results of different studies is generally difficult due to differences in experimental procedures such as the proportion of inoculum used for the experiment phase which has been shown to indeed alter plant performance (Nicot & Rouse, 1987; Pernilla Brinkman, Van der Putten, Bakker, & Verhoeven, 2010), especially as biotic and abiotic conditions may be strong determinants of the strength and direction of feedback (Ehrenfeld et al., 2005; Heinze et al., 2015; Ke, Miki, & Ding, 2015; Mazzoleni et al., 2015; Nicot & Rouse, 1987; Pernilla Brinkman et al., 2010). Further, we used only one species of each functional group for this experiment, making it impossible to distinguish functional group‐specific and species‐specific effects, an issue that could be resolved by a more complex experimental design in future studies.

### 4.3 Can feedback strength be explained by trait dissimilarities?

In our experiment, we did not find a relationship between trait dissimilarities between accession/species or individual traits and the strength of plant–soil feedback, which is in contrast to our third hypothesis that morphology might contribute to feedback effects. It has long been known that soil organisms act as main agents of plant–soil feedback (Bever et al., 1997). In addition, there is a greater awareness that processes such as plant‐mediated nutrient cycling may have impacts on those plant–microbial interactions, thereby contributing to the resulting feedback effects (Ehrenfeld et al., 2005; Teste et al., 2017). In this context, plant functional traits have been shown to have an effect on the abiotic (here: chemical) properties of the soil they are growing in (Binkley & Giardina, 1998). The importance of these plant traits as determinants of plant–soil feedback has recently been shown to depend on the composition of the soil communities (Ke et al., 2015; Mazzoleni et al., 2015). Closely related species may still accumulate distinct soil communities leading to differences in plant performance when growing in different soil types and therefore to different feedback effects (Pendergast, Burke, & Carson, 2013). In contrast to that, other studies have shown that more closely related plant species may be more similar to each other than to distinctly related species with regard to the composition of specific soil communities (Gilbert & Webb, 2007; Webb et al., 2006) as well as in terms of ecological traits such as germination rate, seedling survival (Burns & Strauss, 2011), and responses to infestation by pathogens (Gilbert et al., 2015; Parker et al., 2015). Contrary to our expectations, we did not find such a relationship between feedback strength and species‐specific morphological or functional traits of the involved accessions/species. However, in our study, we only included a limited number of traits. For example, we did not measure possibly relevant belowground traits such as root morphological traits or the ability of plants to influence the composition of soil communities via root exudates, which could clearly have influenced the strength of feedbacks. We also did not consider flowering times or successional stages of the plants, the latter of which has been shown to be connected to the strength of soil feedback (Kardol, Martijn Bezemer, & van der Putten, 2006) This limitation might be part of the reason why we did not find a relationship between traits or trait similarities and feedback strength. A recent study by Cortois, Schröder‐Georgi, Weigelt, van der Putten, and De Deyn (2016) investigated the relationship between feedback strength and several aboveground traits (relative growth rate, specific leaf area) and belowground traits (specific root length, percent arbuscular mycorrhizal fungi colonization) simultaneously and found that the direction and strength of feedback were best explained by the examined belowground traits. Species with high specific root length and low arbuscular mycorrhizal fungi colonization experienced the most negative soil feedback (see also Bennett et al. (2017)). We conclude that future studies that link plant–soil feedback, phylogenetic relatedness, and morphological trait similarities should focus on traits and especially on trait differences in the belowground compartment to better understand the relationship between feedback and phylogeny.

## 5 CONCLUSION

We found that on average, negative plant–soil feedback operated in our experiment, with increasing strength of the negative feedback with increasing taxonomic distance between the involved players. This result has important implications for the assembly and the resulting structure of plant communities. If the taxonomic differences in feedback that we found similarly occur under field conditions, this might, for example, explain why plant communities are more readily invaded by more distantly related species, and why diverse communities are more resistant to invasion (Fargione, Brown, & Tilman, 2003; Petermann et al., 2010). Generally, plant–soil feedback could be an important mechanism maintaining diversity at all taxonomic levels, with stronger structuring effects at high taxonomic levels favoring the highest possible diversity within the limits of the environmental filter. However, negative soil feedback even emerged at the intraspecific level in our experiment, indicating a contribution to the maintenance of diversity even below the species level, where plant–soil feedback is rarely considered as a coexistence mechanism. While we did not find a relationship between plant–soil feedback and absolute traits or trait similarities between accessions/species our results highlight the importance of explicitly considering relatedness when examining plant–soil feedback as a coexistence mechanism.

## CONFLICT OF INTEREST

None declared.

## AUTHOR CONTRIBUTIONS

ARB and JSP designed the experiment. ARB conducted the experiment. ARB, CS, and JSP analyzed the data. ARB wrote the first draft. All authors contributed to writing and revising the manuscript.
