# Supplementary material for: The strength of negative plant–soil feedback increases from the intraspecific to the interspecific and the functional group level
Source: Ecol Evol. 2018 Jan 26;8(4):2280–9. doi: 10.1002/ece3.3755 (PMC5817124; doi:10.1002/ece3.3755)
Supplement: Supplementary file 1 [file ECE3-8-2280-s001.docx]

**Supporting information**


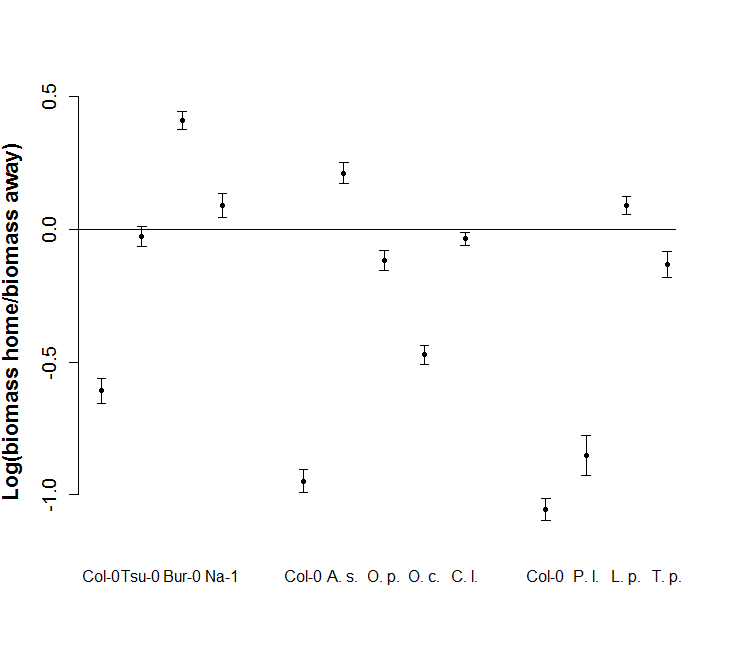


**Fig. S1**. Average bootstrapped plant-soil feedback experienced by the *Arabidopsis thaliana* accessions Col-0, Tsu-0, Bur-0 and Na-1 (intraspecific level) as well as the species *A. suecica* (A. s.), *Olimarabidopsis pumila* (O. p.), *O.* *cabulica* (O. c.), *Crucihimalaya lasiocarpa* (C. l.; interspecific level), P*lantago lanceolata* (P. l.), *Lolium perenne* (L. p.) and *Trifolium* *pratense* (T. p.; functional group level). Feedback values are the log-transformed ratios of the biomass of individual plants on home soils divided by the biomass of individual plants on away soils for each accession/species. Here, the feedback ratios were determined for each accession/species using a bootstrap procedure sampling 1000 times with replacement to test for any effect that the pairing between individuals on home and away soil might have on the results. In addition, 95% bootstrap confidence intervals were calculated. Negative values correspond to a net disadvantage on home soils (negative feedback); positive values to a benefit on home soils (positive feedback).

**Table S1**. Mean trait values of the traits ‘biomass (g)’, ‘height (cm)’ and ‘fitness’ measured during the training phase of the experiment as well as trait values extracted from the literature of the categorical traits ‘rosette’, ‘life form’, ‘association with arbuscular mycorrhizal fungi (AMF)’, ‘nitrogen fixation (NF)’ and ‘life span’ of each accession/species. The trait ‘fitness’ describes the proportion of individuals per pot that produced seeds.

| **accession/species** | **biomass** | **height** | **fitness** | **rosette** | **life form** | **AMF** | **NF** | **life span** |
| --- | --- | --- | --- | --- | --- | --- | --- | --- |
| *Arabidopsis thaliana* Col-0 | 0.14 | 32.72 | 1.00 | yes | herb | no | no | annual |
| *Arabidopsis thaliana* Tsu-0 | 0.19 | 35.61 | 1.00 | yes | herb | no | no | annual |
| *Arabidopsis thaliana* Bur-0 | 0.10 | 15.98 | 0.79 | yes | herb | no | no | annual |
| *Arabidopsis thaliana* Na-1 | 0.11 | 22.98 | 0.89 | yes | herb | no | no | annual |
| *Arabidopsis suecica* | 0.15 | 0.08 | 0.01 | yes | herb | no | no | annual |
| *Olimarabidopsis pumila* | 0.22 | 2.94 | 0.53 | yes | herb | no | no | annual |
| *Olimarabidopsis cabulica* | 0.15 | 5.34 | 0.95 | yes | herb | no | no | annual |
| *Crucihimalaya lasiocarpa* | 0.07 | 20.22 | 0.98 | yes | herb | no | no | annual |
| *Plantago lanceolata* | 0.34 | 19.54 | 0.00 | yes | herb | yes | no | perennial |
| *Lolium perenne* | 0.38 | 38.18 | 0.00 | no | grass | yes | no | perennial |
| *Trifolium pratense* | 0.60 | 20.58 | 0.00 | no | herb | yes | yes | perennial |

**R scripts:**

#mixed-effects models testing the effect of taxonomic level and accession/species identity on feedback(Table 1)

library(nlme)

model1<-lme(feedback_biomass~level_cont+species_precise,random=~1|pot_number,na.action=na.exclude);anova(model1)

#bootstrap procedure testing PSF effects between species/accession and the conditioning species/accession

#example: Col-0 vs. Tsu-0

library(boot)

dfCol0<-Bootstrap[ which(Bootstrap$species_precise=="Col-0"), ]

df1<-subset(dfCol0, cond_species=="Col-0" | cond_species=="Tsu-0",

select=pot_number:aboveground_biomass)

myboot1 <- boot(data=df1, function(x,i){booty1 <-tapply(df1$aboveground_biomass, df1$treatment, FUN=function(x)

sample(na.omit(as.numeric(x)),length(1),TRUE))log(booty1[2]/booty1[1])},

R=1000)

boot.ci(myboot1, type="all")

mean(myboot1$t)

#calculation of trait dissimilarities

library(cluster)

d<-daisy(traitclust,metric=c("gower"))

clust<-agnes(d)

plot(clust)

summary(clust)

#Mantel test testing the relationship between PSF and trait dissimilarities

library(reshape2)

test1<-acast(diss, species~cond_spec, value.var="diss2")

test2<-acast(diss, species~cond_spec, value.var="feedback_biomass.mean")

dist1<-as.dist(test1)

dist2<-as.dist(test2)

library(vegan)

mantel(dist1, dist2, method="pearson", permutations=999, na.rm = TRUE)
